# Triptolide Triggers Protective Autophagy via ROS Induction in NSCLC: Therapeutic Synergy with Autophagy Inhibition

**DOI:** 10.3390/cancers18060902

**Published:** 2026-03-11

**Authors:** Siqi Chen, Mengjia Sun, Quancheng Yang, Yi Lv, Xuejia Zhai

**Affiliations:** 1 Department of Pharmacy, Union Hospital, Tongji Medical College, Huazhong University of Science and Technology, Wuhan 430022, China; m202376311@hust.edu.cn (S.C.); m202476487@hust.edu.cn (M.S.);; 2 Tongji Hospital, Tongji Medical College, Huazhong University of Science and Technology, Wuhan 430030, China

**Keywords:** triptolide, NSCLC, ROS, protective autophagy, chloroquine, combination therapy, oxidative stress, synergistic antitumor effect

## Abstract

**Background:** Triptolide (TPL) is an epoxytriptolide diterpenoid lactone isolated from the traditional Chinese medicinal herb *Tripterygium wilfordii* and exhibits broad pharmacological activities, including anti-inflammatory, immunomodulatory, and antitumor effects. Its water-soluble prodrug, minnelide, is currently undergoing clinical trials for the treatment of pancreatic cancer. Reactive oxygen species (ROS) regulate cellular fate by inducing oxidative damage and activating autophagy, which can promote cell survival under moderate stress but contribute to cell death when excessively or persistently activated. Although TPL has been reported to induce ROS accumulation, its mechanistic role in non-small cell lung cancer (NSCLC) remains incompletely understood. This study aimed to systematically investigate the role of ROS-mediated autophagy in TPL-induced cytotoxicity and to evaluate the therapeutic potential of combining TPL with autophagy inhibition in NSCLC. **Methods:** A series of in vitro experiments was performed to characterize TPL-mediated changes in NSCLC cell proliferation, migration, and ROS production. Autophagy- and apoptosis-related molecular alterations were analyzed using Western blotting and fluorescence microscopy with fluorescent reporter constructs. An H1299 xenograft mouse model was established to assess the antitumor efficacy of TPL in vivo and its combination effects with an autophagy inhibitor. **Results:** In this study, we demonstrated that TPL induces NSCLC cell death primarily through increased ROS levels. Mechanistic analyses further revealed that ROS accumulation simultaneously activates a protective autophagic response. Notably, in vivo experiments showed that co-administration of TPL with the autophagy inhibitor chloroquine resulted in significantly stronger tumor growth suppression than either treatment alone. **Conclusions:** Autophagy acts as a resistance mechanism against TPL-induced cytotoxicity in NSCLC, and pharmacological autophagy inhibition potentiates the antitumor activity of TPL. These findings clarify the ROS–autophagy interplay underlying TPL-mediated cell death and provide a preclinical rationale for combining TPL with autophagy inhibitors as a therapeutic strategy for NSCLC.

## Simple Summary

Non-small cell lung cancer (NSCLC) remains difficult to treat because tumor cells can adapt to therapy and survive. Triptolide, a natural compound from Tripterygium wilfordii, has shown anticancer activity, but its mechanisms in NSCLC are not fully understood. In this study, we found that triptolide increases oxidative stress inside NSCLC cells, which contributes to tumor cell death. At the same time, the cells activate autophagy, a self-protective recycling process that helps them tolerate stress. Importantly, blocking autophagy with chloroquine strengthened the anticancer effect of triptolide in a mouse tumor model. These findings suggest that combining triptolide with autophagy inhibition may be a promising strategy to improve treatment efficacy in NSCLC.

## 1. Introduction

Natural products, particularly traditional Chinese medicine, have emerged as valuable sources of anticancer agents in recent years. Triptolide (TPL), an epoxyditerpenoid lactone derived from the traditional Chinese herb *Tripterygium wilfordii*, exhibits broad pharmacological properties, including anti-inflammatory, immunosuppressive, and antitumor activities [1]. In oncology, TPL exerts broad antitumor effects by inducing apoptosis and cell cycle arrest, suppressing NF-κB-dependent transcription, and modulating stress and survival pathways such as ER stress, MAPK, and PI3K/Akt signaling [2,3,4,5,6]. Notably, translational studies in pancreatic cancer using the water-soluble prodrug minnelide, now in clinical trials, highlight the therapeutic potential of TPL-based strategies [7,8].

Reactive oxygen species (ROS) regulate cancer cell fate in a context-dependent manner, promoting tumor progression at moderate levels but inducing oxidative stress and cell death when excessively accumulated [9,10,11]. Although TPL has been reported to elevate intracellular ROS levels in tumor cells, the precise mechanism of action and subsequent biological effects remain incompletely understood, particularly regarding differential responses across cancer types, which warrant further investigation [12]. Emerging evidence suggests that ROS accumulation not only causes direct cellular damage but may also activate stress-adaptive responses, among which autophagy plays a critical role in determining cell fate [12,13]. Autophagy can be cytoprotective, alleviating ROS-induced stress and promoting tumor cell survival, whereas excessive or prolonged autophagy may induce cell death [14]. Although autophagy plays a multifaceted role in cancer development depending on the tumor stage and cellular microenvironment, it is widely recognized as a protective mechanism in cancer cells that can diminish drug efficacy [14,15]. Accordingly, pharmacological modulation of autophagy, particularly via inhibitors such as chloroquine and hydroxychloroquine, has emerged as a promising strategy to enhance anticancer therapy and is currently being evaluated in clinical trials [16,17,18,19].

As the most prevalent histological subtype of lung cancer, non-small cell lung cancer (NSCLC) accounts for the majority of cases and is responsible for substantial cancer-related mortality globally. Long-term survival remains poor despite advances in targeted therapy and immunotherapy, largely due to frequent development of resistance to chemotherapy and targeted agents. This highlights an urgent need for novel therapeutic strategies and rational drug combinations for NSCLC treatment. Dysregulated ROS signaling and autophagy play pivotal roles in NSCLC initiation, progression, and therapy resistance, suggesting the ROS/autophagy axis as a relevant therapeutic target. Although NSCLC cells are sensitive to TPL-induced growth inhibition, it remains unclear whether ROS-induced autophagy acts as a cytoprotective or cytotoxic mechanism in TPL-treated NSCLC.

In this study, we aimed to comprehensively elucidate the interplay between ROS and autophagy in the antitumor effects of TPL, thereby revealing the full mechanism of action. Using NSCLC cells as a representative model, we investigated whether TPL induces cell death via ROS accumulation and characterized the role of ROS-mediated autophagy. Furthermore, in vivo xenograft experiments evaluated the antitumor efficacy of TPL in combination with the autophagy inhibitor chloroquine. Our findings demonstrate that inhibiting adaptive, protective autophagy markedly enhances TPL-induced cytotoxicity and antitumor activity in vivo. Collectively, these results provide new mechanistic insights into TPL’s pharmacological actions and support combination strategies integrating TPL with autophagy inhibition as a potential therapeutic approach in NSCLC.

## 2. Materials and Methods

### 2.1. Materials

Small-molecule reagents were purchased from MedChemExpress (Monmouth Junction, NJ, USA), including triptolide (HY-32735), Ferrostatin-1 (HY-100579), bafilomycin A1 (HY-100558), SAR405 (HY-12481), rapamycin (HY-10219), Z-VAD-FMK (HY-16658B), N-acetyl-L-cysteine (HY-B0215), necrostatin-1 (HY-15760), Gefitinib (HY-50895), and Chloroquine (HY-17589A). Triptolide was supplied as a 10 mM stock solution in DMSO (solubility in DMSO: ~25 mg/mL, according to the manufacturer) and stored at −20 °C. Stock solutions were freshly diluted in culture medium before use, and the final DMSO concentration did not exceed 0.1%. The ROS Assay Kit (G1706) and JC-1 Mitochondrial Membrane Potential Detection Kit (G1515) were obtained from Servicebio (Wuhan, China). The Annexin V-FITC/PI Cell Apoptosis Detection Kit (E-CK-A211) was obtained from ElabScience (Wuhan, China).

### 2.2. Cell Culture

NSCLC cell lines NCI-H1299 and NCI-H460 were provided by Wuhan Procell Life Science & Technology Co., Ltd. (Wuhan, China) Cells were maintained in RPMI-1640 (Procell, Wuhan, China; PM150110) supplemented with 10% fetal bovine serum (Procell, 164210) and 100 U/mL penicillin–streptomycin (Biosharp, Hefei, China; BL505A) at 37 °C in a humidified incubator with 5% CO_2_. Cell lines were routinely tested and confirmed to be mycoplasma-free.

### 2.3. Detection of Cell Viability

The inhibitory effect of TPL on the proliferation of NSCLC cells was evaluated using the CCK-8 assay. Logarithmic-phase cells were seeded in 96-well plates at a density of 6 × 10^3^ cells/well, with peripheral wells filled with PBS for liquid sealing. After 24 h of adhesion, cells were treated with gradient concentrations of TPL for 24 h. Subsequently, CCK-8 reagent was added, and plates were incubated for 2 h in the dark. Absorbance was measured at 450 nm using a microplate reader. IC_50_ values of TPL against NSCLC cells were calculated using GraphPad Prism 10.0. Experiments were performed in triplicate.

### 2.4. Colony Formation Assay

Cells were seeded at 500 cells/well in 6-well plates and cultured at 37 °C for 24 h. Cells were then treated with a gradient of TPL for 72 h. The drug-containing medium was replaced every three days for approximately 15 days. The cells were then washed with PBS, fixed with paraformaldehyde, stained with 0.1% crystal violet, and washed twice with PBS. Plates were air-dried in the dark and photographed. Colonies containing more than 50 cells were defined as valid clusters and quantified using ImageJ software (version 1.52p).

### 2.5. Transwell and Wound-Healing Assays

For wound-healing assays, logarithmic-phase cells were prepared as a suspension at approximately 5 × 10^4^ cells/mL and seeded in 6-well plates. After cell adhesion and formation of a confluent monolayer, the cells were treated with a drug-containing complete medium. A straight wound was created in the monolayer using a 200 μL pipette tip and cell migration was monitored every 6 h. For Transwell assays, logarithmic-phase cells were prepared as a suspension at approximately 5 × 10^4^ cells/mL and seeded in 6-well plates. After 48 h of treatment with drug-containing complete medium, the cells were trypsinized, resuspended in serum-free medium, and seeded into the upper chamber of a Transwell insert. The lower wells of a 24-well plate were supplemented with 500 μL complete medium. After 48 h of incubation, the Transwell inserts were gently rinsed with PBS, fixed in paraformaldehyde, and stained with 0.1% crystal violet. The membranes were then air-dried in the dark, and migrated cells on the underside were visualized and photographed under a microscope.

### 2.6. Flow Cytometric Apoptosis Analysis

Cells were seeded in 6-well plates, allowed to adhere and treated with the drug for 24 h. Cells were trypsinized, resuspended in binding buffer, and stained with 5 μL Annexin V-FITC and 10 μL PI (20 μg/mL) in the dark for 15 min. Apoptosis rates were analyzed using a Cyto-Flex flow cytometer (Beckman Coulter, Brea, CA, USA). The apoptotic rate was calculated as the percentage of Annexin V-positive cells, including both early apoptotic (Annexin V^+^/PI^−^) and late apoptotic (Annexin V^+^/PI^+^) populations.

### 2.7. Measurement of Intracellular ROS Accumulation and Detection of Mitochondrial Membrane Potential

For ROS detection, cells were seeded in 6-well plates, allowed to adhere, followed by 12 h drug treatment. Cells were incubated with 10 μM DCFH-DA probe for 30 min, washed three times with PBS, and analyzed by flow cytometry to measure relative fluorescence intensity.

Mitochondrial membrane potential (ΔΨm) was evaluated using JC-1 dye. Cells were collected and incubated with JC-1 staining working solution at 37 °C for 20 min. After incubation, the supernatant was discarded and the cells were washed twice with JC-1 staining buffer. ΔΨm was quantified by flow cytometry by measuring JC-1 fluorescence shift from green (JC-1 monomers, low ΔΨm) to red (JC-1 aggregates, high ΔΨm), and the red/green fluorescence intensity ratio was calculated as an indicator of ΔΨm. Carbonyl cyanide m-chlorophenyl hydrazone (CCCP) was used as a positive control for ΔΨm dissipation.

### 2.8. Western Blotting

Following drug treatment, cells were lysed in RIPA buffer (Biosharp, Hefei, China; BL504A). Protein concentrations were quantified using a BCA assay (Biosharp, BL1054A), and sample loading volumes were adjusted accordingly. Equal protein amounts were separated by SDS-PAGE, transferred to PVDF membranes (MerckMillipore, Tullagreen, Carrigtwohill, Co. Cork, Ireland; IPVH00010), and blocked with 5% skim milk. Membranes were incubated with primary antibodies against β-Actin (Servicebio, Wuhan, China; GB15003-100; 1:5000), LC3B (STARTER, Hangzhou, China; S0B0404; 1:1000), and SQSTM1/P62 (STARTER, S0B0586; 1:1000), followed by horseradish peroxidase (HRP)-conjugated goat anti-rabbit secondary antibody (STARTER, S0B4002) for 1 h at room temperature. Protein-antibody complexes were detected using an electrochemiluminescence (ECL) detection kit (Med Chem Express, Monmouth Junction, NJ, USA; HY-K1005).

### 2.9. Generation of Stable Cell Lines

To establish stable cell lines expressing either the RFP-GFP-LC3B or GFP-LC3B reporter, recombinant lentiviruses encoding stubRFP-senseGFP-LC3B or GFP-LC3B were generated by Shanghai GeneChem (Shanghai, China). Stable cell lines were subsequently developed using lentivirus-mediated transduction. H1299 cells were seeded to reach ~20–30% confluence and then transduced with lentiviral particles (1 × 10^8^ TU/mL; 10 μL per well) in the presence of P reagent (8 μL per well; ~1 × 10^5^ cells). Seventy-two hours after transduction, puromycin was applied to the culture medium to select stably transduced cells.

### 2.10. Animal Treatment

Five-week-old BALB/c nude mice (n = 5 per group) were purchased from Hubei Bioent Biological Technology Co., Ltd. (Wuhan, China). Animals were maintained under specific pathogen-free (SPF) conditions at the Experimental Animal Center, Institute of Medicine, Tongji Medical School (Hubei, China). H1299 cells (1 × 10^6^) were injected subcutaneously into the right scapula. Tumor volume was calculated as V = (A × B^2^)/2 mm^3^. When tumor volumes reached 50–100 mm^3^, nude mice were randomized into groups and administered via intraperitoneal injection. The control group received physiological saline every two days. The dosing regimen of TPL was determined based on previous literature and preliminary tolerability experiments. When tumor volumes in control group reached approximately 1500 mm^3^, all mice were euthanized via anesthesia with pentobarbital sodium. Tumor tissues and major organs, including the heart, liver, kidneys, spleen, and lungs, were collected. Half of each tumor tissue sample was sectioned for hematoxylin and eosin (HE) staining. All animal experiments were approved by the Animal Care and Use Committee of Huazhong University of Science and Technology (Approval No. 4981). H1299 cells were selected for the xenograft experiments as they were used for the majority of mechanistic analyses and exhibited reproducible tumorigenicity in nude mice.

## 3. Results

### 3.1. TPL Inhibits Proliferation and Migration of NSCLC Cell Lines

**Figure 1:**
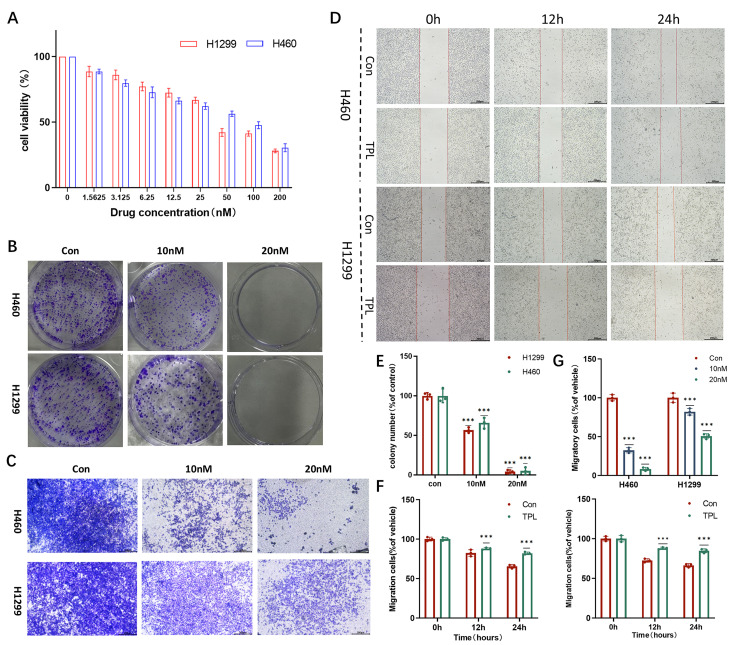
TPL inhibits cell proliferation in NSCLC cells. (**A**) The effects of TPL (0–200 nM, 24 h) on cell viability in H1299 and H460 cells were evaluated by CCK-8 assay (n = 3). (**B**,**E**) Colony formation assay was used to evaluate the anti-proliferative effect of H1299 and H460 cells treated with the TPL (10 nM, 20 nM). (**C**,**G**) Transwell assay was used to detect the invasion of the H1299 and H460 cells treated with the TPL (10 nM, 20 nM) for 24 h. (**D**,**F**) The wound healing assay was used to detect the inhibitory effect of TPL on the migration of H1299 and H460 cells. Data were shown as mean ± SD from at least three independent experiments. The levels of significance were obtained by comparing with the control group (*** *p* < 0.001).

To evaluate the cytotoxic and growth-inhibitory effects of TPL on NSCLC cells, the human NSCLC cell lines H1299 and H460 were treated with TPL (Figure 1). Cell viability was analyzed using the CCK-8 assay following 24 h treatment with increasing concentrations of TPL. TPL significantly inhibited the growth of both H1299 and H460 cells in a dose-dependent manner (Figure 1A). Based on nonlinear regression analysis of the dose–response curves, the IC_50_ values of TPL were calculated to be 48.94 nM for H1299 cells and 61.54 nM for H460 cells. The antiproliferative effect of TPL was further assessed by colony formation assays. TPL treatment markedly reduced colony formation in both cell lines in a dose-dependent manner (Figure 1B,E), confirming sustained inhibition of cell proliferation by TPL. Given that cell motility and invasiveness are fundamental steps in the metastatic cascade, we subsequently investigated the impact of TPL on the migratory capacity of H1299 and H460 cells. Wound-healing assays demonstrated that TPL treatment significantly impaired the migratory ability of H1299 and H460 cells compared with untreated controls (Figure 1D,F). Transwell assays further confirmed that TPL significantly suppressed both migration and invasion of these cell lines (Figure 1C,G).

### 3.2. TPL Exerts Antitumor Efficacy In Vivo

**Figure 2:**
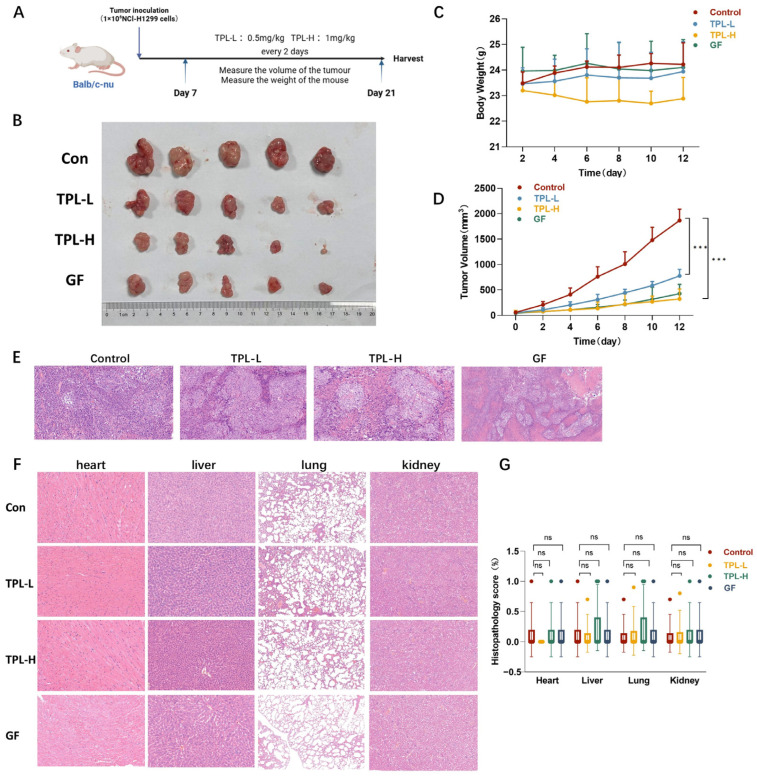
TPL exerts antitumor efficacy in vivo. (**A**) BALB/c nude mice were subcutaneously inoculated with H1299 cells to establish a xenograft tumor model, followed by intraperitoneal administration with low-dose TPL (0.5 mg/kg, TPL-L), high-dose TPL (1 mg/kg, TPL-H), gefitinib (GF), or physiological saline (Control, Con) once every two days (n = 5). (**B**) Representative images of xenograft tumors derived from H1299 cells. (**C**) Triptolide treatment does not change the body weight of mice during the whole animal work. (**D**) Tumor growth curves indicated that TPL markedly suppressed the progression of H1299 xenografts. (**E**) H&E staining of tumors. (**F**,**G**) Histopathological evaluation of heart, lung, kidney, and liver tissues from control (Con), gefitinib (GF), and TPL-treated mice (n = 5) was performed using H&E staining. Data was shown as mean ± SD. The levels of significance were obtained by comparing with the control group (ns, *p* ≥ 0.05; *** *p* < 0.001).

To evaluate the antitumor efficacy of TPL against NSCLC tumor growth in vivo, a subcutaneous xenograft model was established by inoculating H1299 cells into BALB/c nude mice. Seven days post-implantation, mice were randomly assigned to four groups: control, low-dose TPL (TPL-L), high-dose TPL (TPL-H), and a positive control group treated with gefitinib (GF). Saline or the corresponding doses of TPL were administered via intraperitoneal injection every other day for two weeks (Figure 2A). Throughout the treatment, no obvious changes in body weight were observed between control and TPL-L-treated mice (Figure 2C). Notably, both low- and high-dose TPL significantly inhibited tumor growth in a dose-dependent manner, as reflected by reduced tumor volumes compared with controls (Figure 2B,D).

Histopathological evaluation of tumor tissues by H&E staining was performed on tumor tissues from treated mice to evaluate morphology and assess drug efficacy. In the control group, tumor sections showed densely packed malignant cells with high cellularity, increased nuclear-to-cytoplasmic ratios, and prominent nuclear hyperchromasia, consistent with active tumor growth. In contrast, tumors from the TPL-treated groups (TPL-L and TPL-H) and the gefitinib-treated group exhibited pronounced morphological and architectural alterations, including reduced cell density, disrupted tissue architecture, apparent necrosis, vacuolation, and focal inflammatory cell infiltration (Figure 2E).

To evaluate potential systemic toxicity of TPL in vivo, major organs (heart, liver, kidney, and lungs) were harvested, paraffin-embedded, sectioned, and subjected to H&E staining. No obvious histopathological abnormalities were observed in these organs from TPL-treated groups compared with controls (Figure 2F,G).

### 3.3. TPL Induces ROS Accumulation, Triggering Apoptosis

**Figure 3:**
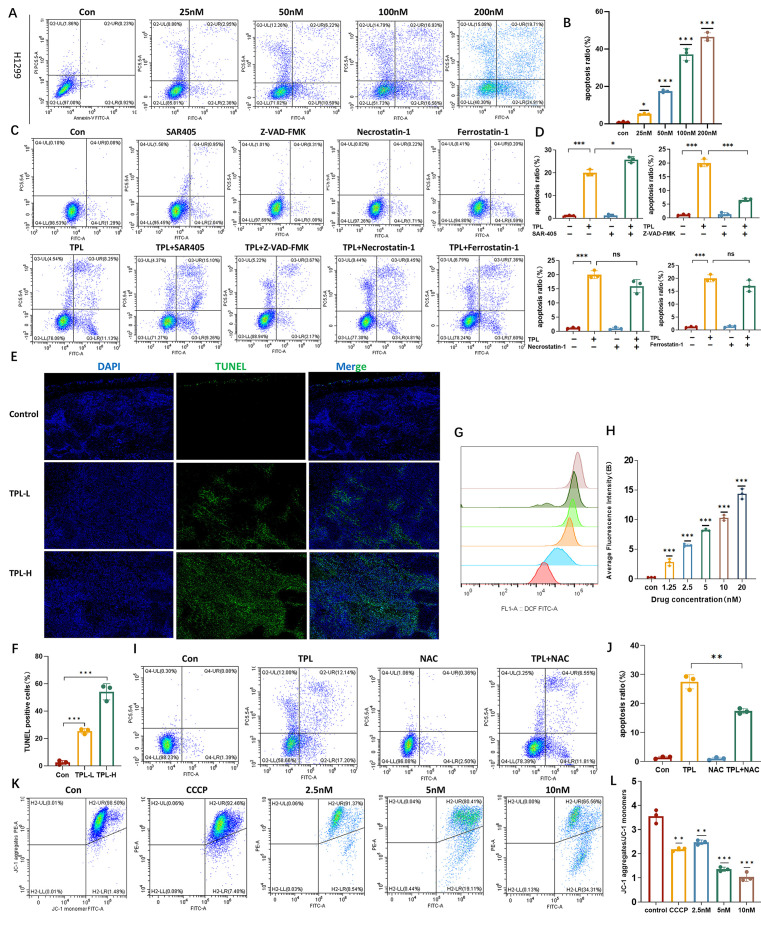
TPL induces ROS accumulation, triggering cellular apoptosis. (**A**,**B**) Cell viability was assessed by flow cytometry in H1299 cells following treatment with gradient concentrations of TPL for 24 h. (**C**,**D**) Pharmacological inhibitor panel analysis in H1299 cells exposed to TPL (100 nM, 24 h) (n = 3). (**E**,**F**) Representative TUNEL images (green) and quantification of apoptotic cells in sections from H1299 xenograft tumors in control and TPL-treated mice (n = 3). (**G**,**H**) Intracellular ROS levels were assessed in H1299 cells following TPL treatment (100 nM, 24 h) (n = 3). (**I**,**J**) ROS inhibitor NAC (N-Acetylcysteine) rescues TPL-induced apoptosis induced by TPL. (**K**,**L**) JC-1 fluorescent probe was used to measure mitochondrial membrane potential (ΔΨm) in NCI-H1299 cells treated with TPL (2.5 nM–10 nM, 24 h). Data was shown as mean ± SD. The levels of significance were obtained by comparing with the control group (ns, *p* ≥ 0.05, * *p* < 0.05, ** *p* < 0.01, *** *p* < 0.001).

To determine whether TPL induces apoptosis in NSCLC cells, apoptosis rates were measured after 24 h treatment with increasing concentrations of TPL. TPL treatment significantly reduced cell viability and increased apoptosis in H1299 cells, demonstrating a potent pro-apoptotic effect on NSCLC cells even at nanomolar concentrations (Figure 3A,B). To elucidate the mechanism underlying TPL-induced NSCLC cell death, cells were co-treated with TPL and inhibitors targeting apoptosis (ferrostatin-1), autophagy (SAR405), or necroptosis (necrostatin-1). Among these, only pan-caspase inhibitor Z-VAD-FMK markedly attenuated TPL-induced cell death, indicating that apoptosis is the predominant mode of cytotoxicity (Figure 3C,D). These results indicate that apoptosis is the predominant mechanism of TPL-mediated cytotoxicity in NSCLC cells. Consistent with the in vitro findings, TPL-induced apoptosis was further confirmed in vivo using a subcutaneous tumor model. TUNEL staining of H1299-derived tumors demonstrated a marked increase in apoptotic cell numbers in TPL-treated mice compared with control animals (Figure 3E,F). Intracellular ROS levels were subsequently quantified by dichlorodihydrofluorescein diacetate (DCFH-DA) staining. TPL exposure resulted in a robust and concentration-dependent elevation of intracellular ROS levels in NSCLC cells (Figure 3G,H).

Since elevated ROS levels are known to induce apoptosis, the ROS inhibitor, *N*-acetylcysteine (NAC), was used to determine whether ROS accumulation drives TPL-induced apoptosis. NAC treatment significantly attenuated TPL-induced cell death in H1299 cells (Figure 3I,J), indicating that ROS accumulation is a critical mediator of TPL-mediated NSCLC cell death. Finally, to determine whether TPL-induced apoptosis involves the mitochondrial pathway, mitochondrial membrane potential (ΔΨm) in NCI-H1299 cells was assessed using the JC-1 probe. Compared with the untreated control group, TPL treatment resulted in a dose-dependent mitochondrial depolarization, characterized by a significant decrease in ΔΨm (Figure 3K,L). Together, these results demonstrate that TPL induces ROS-dependent mitochondrial apoptosis in NSCLC cells.

### 3.4. TPL Engages an Autophagy-Related Stress Response

**Figure 4:**
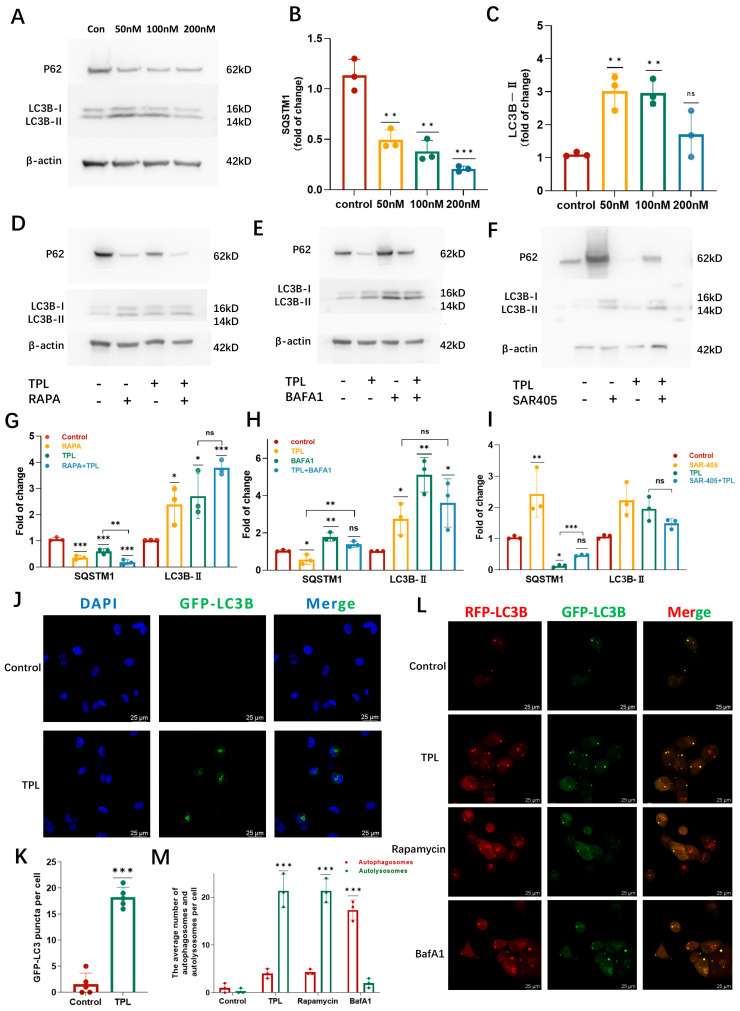
TPL modulates autophagy in NSCLC cells. (**A**–**C**) TPL induces the expression of autophagy markers. Relative levels of autophagy markers SQSTM1/P62 and LC3B-II in H1299 cells treated with the indicated concentration of TPL for 24 h were detected by Western blotting (n = 3). LC3B-I and LC3B-II are indicated on the blot; the lower band corresponds to lipidated LC3B-II. The uncropped blots and molecular weight markers are provided in Supplementary Figure S1A. (**D**–**I**) Immunoblotting was performed to assess LC3B-II and SQSTM1/p62 levels in H1299 cells treated with PBS or TPL (100 nM), with or without rapamycin (Rapa, 250 nM), bafilomycin A1 (BafA1, 1 μM), or SAR405 (10 μM) for 2 h. Band densities were quantified by densitometry (n = 3). The uncropped blots and molecular weight markers are provided in Supplementary Figure S1D–F. (**J**,**K**) H1299 cells stably expressing GFP-LC3 were treated with triptolide for 24 h and subsequently analyzed by confocal microscopy for immunofluorescence. (**L**,**M**) H1299 cells stably expressing GFP-RFP-LC3 were treated with TPL, rapamycin, or bafilomycin for 24 h and subsequently subjected to immunofluorescence analysis using confocal microscopy. Data was shown as mean ± SD. The levels of significance were obtained by comparing with the control group (ns, *p* ≥ 0.05, * *p* < 0.05, ** *p* < 0.01, *** *p* < 0.001).

LC3 (microtubule-associated protein 1 light chain 3) and SQSTM1/p62 are commonly used markers of autophagy. Immunoblot analysis showed that TPL treatment increased LC3B-II levels and reduced SQSTM1/p62 expression, indicating activation of an autophagy-related stress response. However, LC3B-II accumulation did not scale linearly with increasing TPL concentrations, suggesting a context-dependent modulation of autophagy (Figure 4A–C). To further examine the impact of TPL on autophagy-associated pathways, cells were co-treated with the autophagy inducer rapamycin (Rapa), early-stage autophagy inhibitor SAR405, or late-stage autophagy inhibitor bafilomycin A1 (BafA1). Rapa further enhanced the TPL-induced changes in SQSTM1/p62 expression (Figure 4D–I). In contrast, SAR405 partially restored SQSTM1/p62 levels, whereas the LC3B-II increase did not reach statistical significance under these conditions. Notably, co-treatment with TPL and BafA1 did not yield the typical additional LC3B-II accumulation expected under conditions of robust autophagic flux (Figure 4E), suggesting a context-dependent and potentially non-canonical modulation of autophagy-associated pathways by TPL. Collectively, these biochemical data indicate that TPL alters autophagy-associated markers and engages an autophagy-related stress response; however, changes in LC3B-II and SQSTM1/p62 alone are insufficient to conclusively establish a marked increase in canonical autophagic flux under the experimental conditions used.

To further characterize TPL-induced autophagy-associated vesicle dynamics, NCI-H1299 cells stably expressing GFP–LC3 were examined by confocal microscopy. TPL treatment induced a pronounced increase in LC3-positive puncta, consistent with enhanced formation and/or accumulation of LC3-labelled vesicular structures (Figure 4J,K). To assess vesicle maturation, cells expressing the tandem mRFP–GFP–LC3 reporter via lentiviral transduction were analyzed. In this reporter system, GFP fluorescence is quenched in acidic lysosomal environment, such that yellow (GFP+RFP+) puncta represent autophagosomes, whereas red-only (GFP−RFP+) puncta indicate autolysosomes. TPL treatment markedly increased the number of red-only puncta (Figure 4L,M), suggesting enhanced autophagy-associated vesicle maturation/acidification. As expected, the positive control Rapa increased autolysosome formation, whereas the autophagy inhibitor BafA1 (negative control) caused the accumulation of autophagosome-associated signals, validating the blockade of late-stage autophagy. These findings indicate that TPL activates an autophagy-associated stress response rather than robust canonical autophagic flux.

### 3.5. Autophagy Exerts a Cytoprotective Role Under TPL Treatment

**Figure 5:**
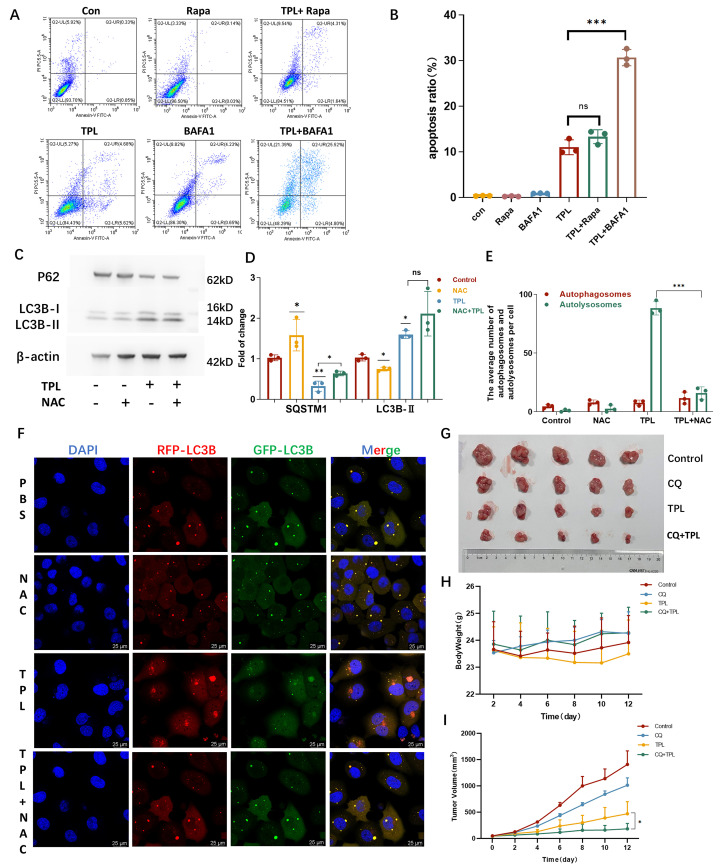
Autophagy promotes cell survival under triptolide treatment. (**A**,**B**) Autophagy inhibitor BAFA1 increases apoptosis ratio in TPL-treated H1299 and H460 cells. Apoptosis was detected by annexin V-FITC/PI staining through flow cytometry analysis. (**C**,**D**) ROS inhibitor NAC effectively rescues TPL-induced reduction in SQSTM1/P62 and LC3B-II levels. The uncropped blots and molecular weight markers are provided in Supplementary Figure S2C. (**E**,**F**) NAC inhibits TPL-induced autophagy flux. NSCLC cells were treated with NAC together with TPL for 24 h, before RFP-GFP-LC3 assay was performed to detect the number of puncta (**F**), which was quantified in (**E**). (**G**) Image of H1299-derived subcutaneous tumors treated with physiological saline, TPL (1 mg/kg), CQ (40 mg/kg), or the combination (TPL 1 mg/kg + CQ 40 mg/kg) (n = 5 mice per group). (**H**) TPL treatment does not change the body weight of mice during the whole animal work. (**I**) Tumor volume curves showed that TPL + CQ combination therapy exhibited significantly stronger suppression of tumor growth compared to TPL alone. Data was shown as mean ± SD. The levels of significance were obtained by comparing with the control group (ns, *p* ≥ 0.05, * *p* < 0.05, ** *p* < 0.01, *** *p* < 0.001).

To determine the functional role of autophagic flux in TPL-induced cytotoxicity, H1299 cells were co-treated with TPL and either the autophagy inducer Rapa or the late-stage autophagy inhibitor BafA1. Pharmacological inhibition of autophagy significantly enhanced TPL-induced apoptosis (Figure 5A,B), indicating that TPL-induced autophagic flux is predominantly cytoprotective in NSCLC cells.

We subsequently examined the relationship between TPL-induced autophagy and ROS production. In H1299 cells, co-treatment with the ROS inhibitor NAC effectively reversed the TPL-induced decrease in SQSTM1/p62 levels (Figure 5C,D), suggesting that ROS accumulation is required for activation of TPL-triggered autophagic flux. Consistently, fluorescence staining confirmed that NAC significantly reduced the TPL-induced increase in red-only puncta (Figure 5E,F), further supporting a role for ROS as an upstream regulator of TPL-mediated autophagic flux.

To evaluate whether the combination of the autophagy inhibitor CQ with TPL enhances antitumor efficacy in vivo, a subcutaneous xenograft model was established using H1299 cells, and tumor-bearing mice were randomized into four treatment groups: vehicle control, TPL monotherapy, CQ monotherapy, and TPL + CQ combination therapy. Throughout the treatment period, no significant differences in body weight were observed among the groups (Figure 5H), indicating favorable tolerability of the treatments. Notably, the combination group exhibited significantly greater tumor growth inhibition than TPL alone, as reflected by a marked reduction in tumor volume (Figure 5G,I). These findings demonstrate that inhibition of autophagy markedly potentiates the antitumor activity of TPL in vivo, supporting the therapeutic potential of combining TPL with autophagy-targeting agents. Collectively, these data establish ROS-induced autophagy as a cytoprotective mechanism that limits TPL antitumor efficacy.

## 4. Discussion

Here, we show that TPL induces NSCLC cell death in association with ROS accumulation. Simultaneously, it activates an autophagy-associated stress response that functionally antagonizes apoptosis. Importantly, pharmacological inhibition of late-stage autophagy—using bafilomycin A1 (BafA1) in vitro and chloroquine (CQ) in vivo—significantly enhanced the antitumor efficacy of TPL, supporting the rationale for combining TPL with autophagy inhibition as a potential therapeutic strategy for NSCLC.

Elevated ROS accumulation is a well-established trigger of cell death through multiple mechanisms, including mitochondrial dysfunction and activation of oxidative stress-responsive signaling pathways [20]. Consistent with previous studies showing that TPL suppresses tumor progression across diverse cancer types by inducing ROS accumulation, our findings indicate that ROS generation is a key mechanism underlying TPL-triggered cytotoxicity [21,22,23]. The current study demonstrates that TPL increases intracellular ROS levels in NSCLC cells. Moreover, ROS scavenging markedly attenuated TPL-induced apoptotic responses, supporting a causal role for ROS in this context. It should be noted that intracellular ROS levels in the present study were mainly detected using the DCFH-DA probe, which reflects overall oxidative stress but does not distinguish individual ROS, such as superoxide or hydrogen peroxide. Importantly, TPL treatment also resulted in a significant decrease in mitochondrial membrane potential, indicating mitochondrial dysfunction and suggesting the involvement of mitochondrial-derived ROS. Together with the protective effect of NAC, these findings imply that mitochondrial superoxide and its downstream metabolite hydrogen peroxide may contribute to TPL-induced oxidative stress signaling in NSCLC cells. However, ROS elevation is also known to activate stress-adaptive responses, particularly autophagy, which together with apoptosis critically determines cell fate and influences therapeutic efficacy in NSCLC and other malignancies [24,25]. Further studies using specific probes, such as MitoSOX and HyPer, will be required to precisely characterize the ROS species involved in TPL-induced cytotoxicity.

In addition to Annexin V/PI staining and mitochondrial membrane potential analysis, the protective effect of the pan-caspase inhibitor Z-VAD-FMK indicates that TPL-induced cell death is predominantly caspase-dependent. Given that mitochondrial dysfunction represents a key upstream event in intrinsic apoptosis [26], it is plausible that pro-apoptotic Bcl-2 family members, such as Bax and Bak, as well as downstream caspase-9 and caspase-3 activation, contribute to TPL-induced cytotoxicity. Although these molecular events were not directly examined in the present study, the observed mitochondrial depolarization and caspase inhibition data collectively support activation of the intrinsic apoptotic pathway. Further mechanistic studies are warranted to delineate the precise apoptotic signaling cascade.

A key finding of this study is the functional characterization of TPL-induced autophagy. While TPL altered LC3 processing and increased autophagy-associated vesicle formation, LC3-II accumulation alone cannot define autophagic function. Instead, pharmacological inhibition of late-stage autophagy significantly potentiated TPL-induced apoptosis, indicating that the autophagic response serves predominantly as an adaptive survival mechanism. Thus, our findings functionally define TPL-induced autophagy as cytoprotective rather than pro-death in NSCLC cells. Upstream regulation of this process likely involves ROS-responsive signaling pathways. Autophagy initiation is tightly controlled by stress-sensing modules such as the AMPK–mTOR–ULK1 axis [27,28,29]. Under oxidative stress, ROS can activate AMPK and suppress mTORC1, thereby promoting ULK1-dependent autophagosome formation [30]. In addition, ROS-mediated JNK activation may disrupt the Beclin1–Bcl-2 complex, further facilitating autophagy initiation [24,31]. Although these pathways were not directly examined, the convergence of ROS accumulation, mitochondrial dysfunction, and autophagic activation strongly supports a ROS-driven autophagy model in TPL-treated NSCLC cells.

From a translational perspective, CQ disrupts lysosome-dependent stress adaptation by inhibiting lysosomal acidification, thereby preventing completion of the autophagic process and promoting the accumulation of damaged organelles and oxidative stress signals. This mechanism provides a clear rationale for the enhanced antitumor efficacy observed with the TPL–CQ combination. In vivo, the combination regimen produced significantly greater tumor growth suppression than either agent alone, without overt toxicity under the dosing schedule used in this study. Nevertheless, more comprehensive toxicological evaluations—including serum biochemistry, histopathological analysis of major organs, and longer follow-up—will be required before clinical translation can be considered.

Although the TPL–CQ combination demonstrated favorable tolerability in vivo, known toxicities associated with both agents warrant careful dose optimization and safety monitoring. TPL is known to cause dose-limiting hepatotoxicity, nephrotoxicity, cardiotoxicity, and myelosuppression, while prolonged exposure to CQ and other late-stage autophagy inhibitors may lead to retinopathy, cardiomyopathy, and gastrointestinal or hematological adverse events. To mitigate these risks, future studies should focus on: (i) optimizing dosing schedules and treatment durations to achieve maximal antitumor efficacy at the lowest effective doses; (ii) evaluating emerging autophagy inhibitors with improved selectivity and safety profiles; and (iii) developing rational delivery strategies (e.g., tumor-targeted or lung-directed formulations of TPL to enhance intratumoral drug accumulation while reducing systemic exposure. In addition, careful clinical monitoring of liver, kidney, and cardiac function, along with regular ophthalmologic examination in patients receiving chloroquine-based regimens, will be essential to avoid or promptly manage treatment-related toxicities. These considerations underscore that while our preclinical data support the promise of TPL–autophagy inhibitor combinations, their safe clinical implementation will require judicious dose and schedule optimization, together with rigorous toxicity surveillance.

Collectively, our findings deepen the understanding of TPL’s antitumor mechanisms and highlight autophagy modulation as a promising strategy for overcoming therapy resistance in cancer. This approach is particularly relevant for NSCLC, where resistance to conventional chemotherapy and targeted therapies remains a major challenge, underscoring the importance of developing novel combination therapies based on natural products. Although TPL demonstrates promising anticancer efficacy, its clinical translation has been limited by poor aqueous solubility, a narrow therapeutic window, and multi-organ toxicity [32]. To address these limitations, water-soluble prodrugs of TPL (e.g., phosphate-modified derivatives) and nanotechnology-based delivery systems, including liposomes, polymeric micelles, and lipid–polymer hybrid nanoparticles, have been developed to improve its solubility, systemic exposure, and tumor-targeting. These formulation strategies are expected to facilitate clinical translation of TPL-based regimens for NSCLC by enabling safer and more controllable doses. Recent advances have indicated that novel strategies, such as nanoformulations, prodrug development, and metabolic engineering, further support the feasibility of enhancing tissue-specific accumulation while minimizing off-target toxicity [8,33]. In summary, this study identifies ROS-associated, cytoprotective autophagy as a critical determinant of TPL responsiveness in NSCLC. Pharmacological inhibition of autophagy significantly enhances TPL antitumor efficacy, providing a strong rationale for combination therapy. These findings support further translational development of TPL-based regimens, with emphasis on optimized delivery, dosing, and safety evaluation.

## 5. Conclusions

Our study demonstrates that TPL suppresses NSCLC growth by inducing intracellular ROS accumulation. However, TPL simultaneously activates a protective autophagic response that partially mitigates its cytotoxic effects. Notably, combination therapy with the autophagy inhibitor chloroquine significantly enhanced the antitumor efficacy of TPL and was well tolerated in vivo, supporting a rational, clinically relevant combination strategy to potentiate ROS-driven tumor cell death in NSCLC. Collectively, these findings highlight the translational potential of targeting the ROS–autophagy axis to improve therapeutic responses and potentially overcome treatment resistance in NSCLC. Future studies should validate this combination in more clinically relevant models (e.g., patient-derived xenografts and organoids), systematically define optimal dosing schedules and safety profiles, and investigate mechanisms of adaptive resistance to TPL–autophagy inhibition to facilitate clinical translation.

## Data Availability

All data supporting this study are included in the article and its Supplementary Materials.

## Supplementary Materials

The following supporting information can be downloaded at: https://www.mdpi.com/article/10.3390/cancers18060902/s1, Figure S1A. Uncropped Western blots with molecular weight markers corresponding to Figure 4A; Figure S1D–F. Uncropped Western blots with molecular weight markers corresponding to Figure 4D–F; Figure S2C. Uncropped Western blots with molecular weight markers corresponding to Figure 5C.

## Abbreviations

TPL	Triptolide
ROS	reactive oxygen species
non-small cell lung cancer	NSCLC
Keap1	Kelch-like ECH-associated protein-1
Nrf2	Nuclear factor erythroid 2-related factor 2
CCK-8	Cell Counting Kit-8
Z-VAD-FMK	Benzyloxycarbonyl-Val-Ala-Asp(OMe)-fluoromethylketone
DCFH-DA	2′,7′-dichlorofluorescin diacetate
NAC	N-acetyl-cysteine
GF	gefitinib
SQSTM1/p62	sequestosome 1
LC3	microtubule-associated protein 1 light chain 3
H&E	hematoxylin and eosin
GFP	Green fluorescent protein
RFP	Red fluorescent protein
RAPA	Rapamycin
BAFA1	bafilomycin A1
CQ	chloroquine
NF-κB	nuclear factor kappa-B
MAPK	mitogen-activated protein kinase
PI3K	Phosphatidylinositol 3-kinase
Akt	protein kinase B
