# Supplementary material for: Triptolide Triggers Protective Autophagy via ROS Induction in NSCLC: Therapeutic Synergy with Autophagy Inhibition
Source: Cancers (Basel). 2026 Mar 11;18(6):902. doi: 10.3390/cancers18060902 (PMC13024899; doi:10.3390/cancers18060902)
Supplement: Supplementary file 1 [file cancers-18-00902-s001.zip › cancers-4148612-supplementary.pdf]

Fig S4-A ①

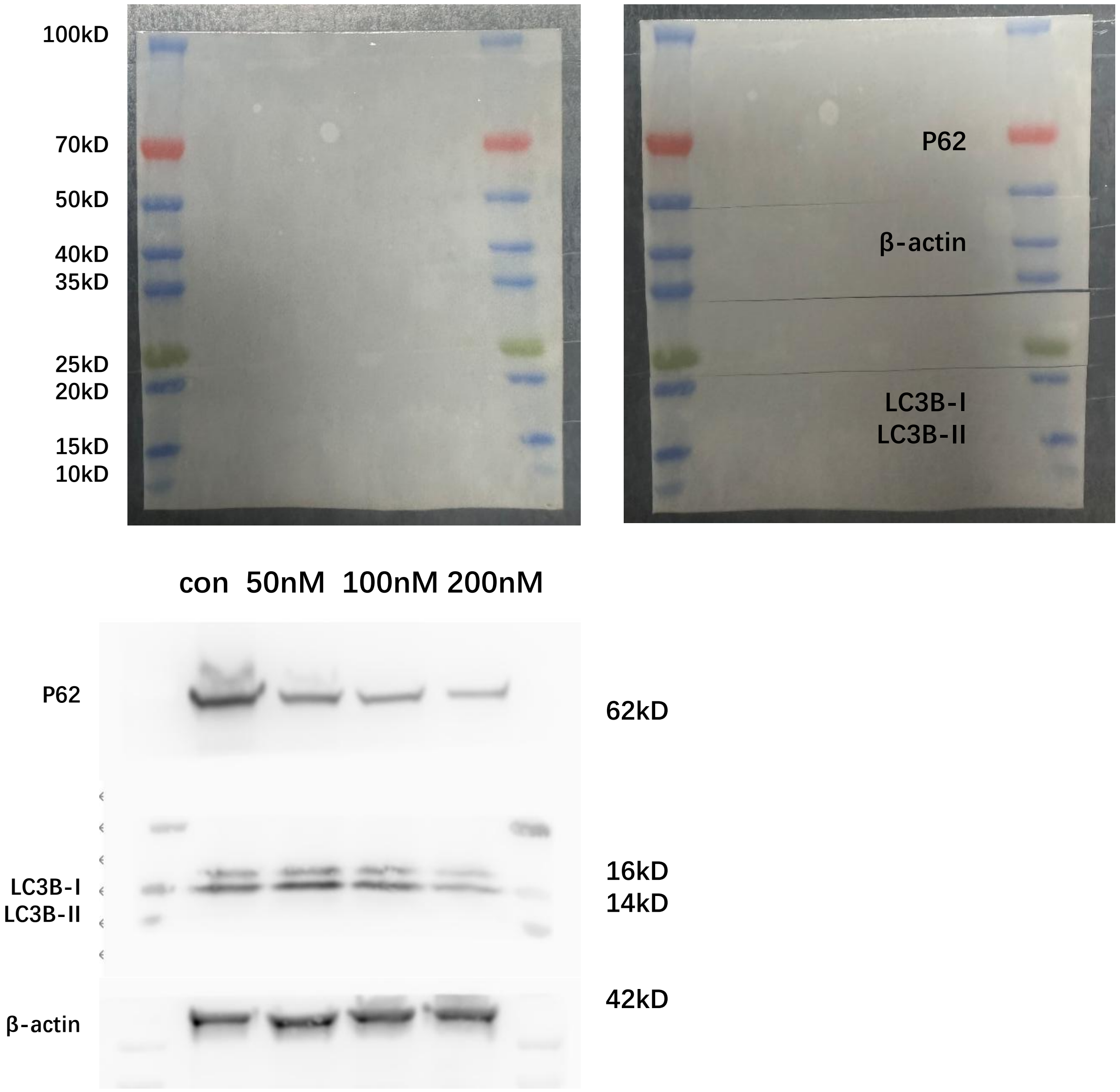

100kD
70kD
50kD
40kD
35kD
25kD
20kD
15kD
10kD
P62
β-actin
LC3B-I
LC3B-II
con
50nM
100nM
200nM
P62
62kD
LC3B-I
LC3B-II
16kD
14kD
42kD
β-actin

Fig S4-A ②③

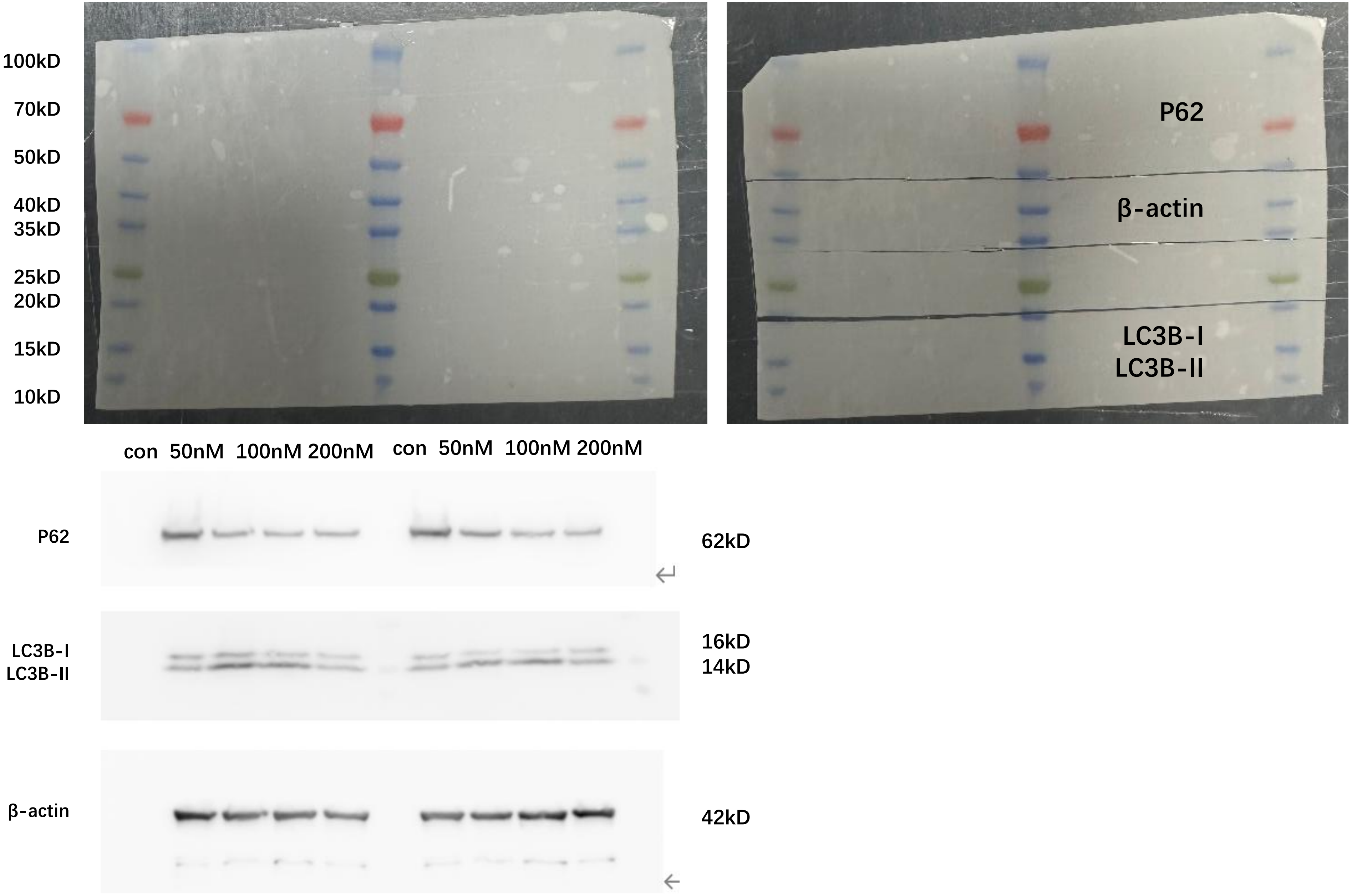
100kD
70kD
50kD
40kD
35kD
25kD
20kD
15kD
10kD
P62
β-actin
LC3B-I
LC3B-II
con 50nM 100nM 200nM con 50nM 100nM 200nM
P62
62kD
LC3B-I
LC3B-II
16kD
14kD
β-actin
42kD

Fig S4-D E ①

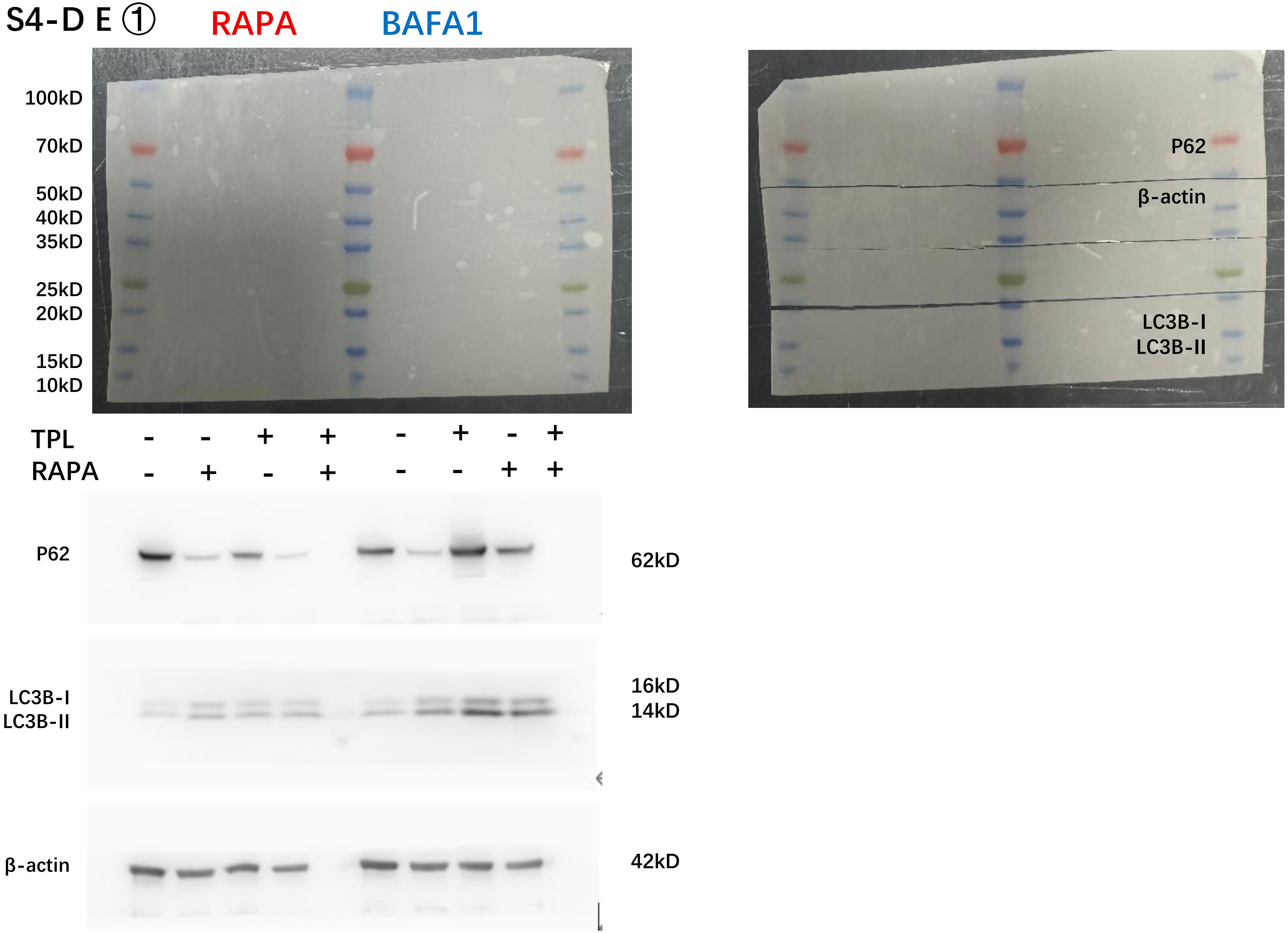
RAPA
BAFA1
100kD
70kD
50kD
40kD
35kD
25kD
20kD
15kD
10kD
P62
β-actin
LC3B-I
LC3B-II
TPL - - + + - + - +
RAPA - + - + - - + +
P62
62kD
LC3B-I
LC3B-II
16kD
14kD
β-actin
42kD

Fig S4-D E ②

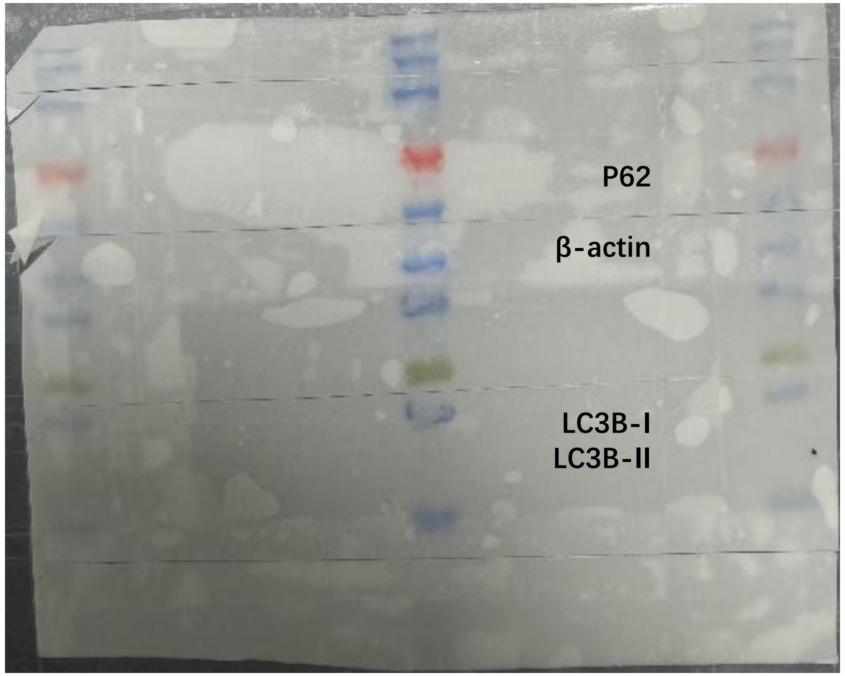
RAPA
BAFA1
100kD
70kD
50kD
40kD
35kD
25kD
20kD
15kD
P62
β-actin
LC3B-I
LC3B-II
TPL - - + + - + - + TPL
RAPA - + - + - - + + BAFA1

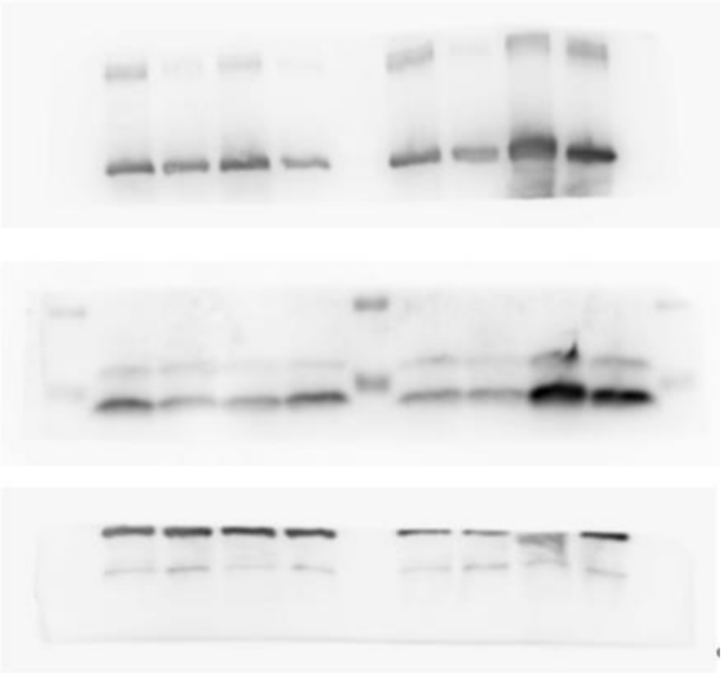
RAPA
BAFA1
P62
LC3B-I
LC3B-II
β-actin
TPL - - + + - + - + TPL
RAPA - + - + - - + + BAFA1

Fig S4-D E ③

BAFA1

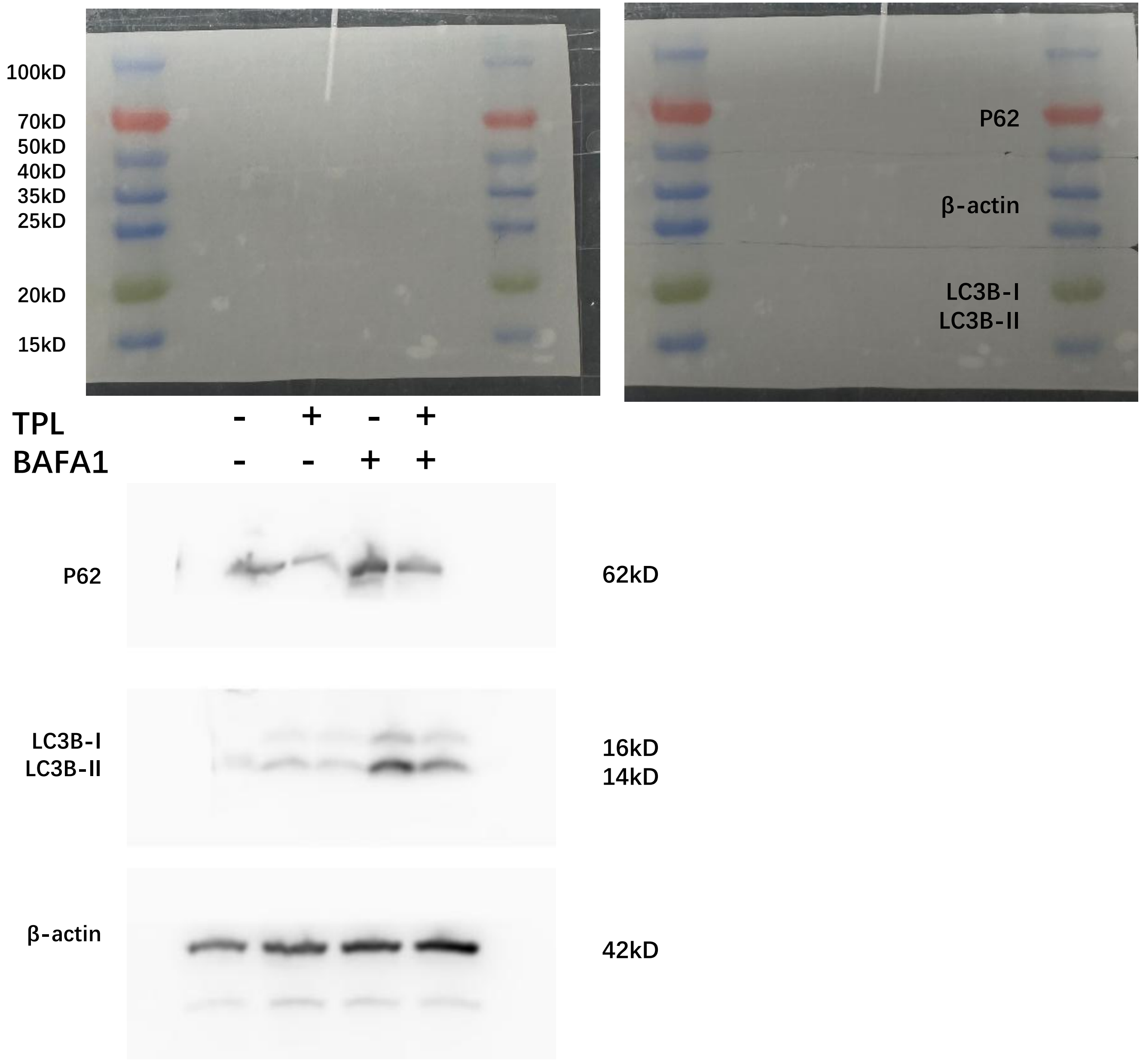

Rapa

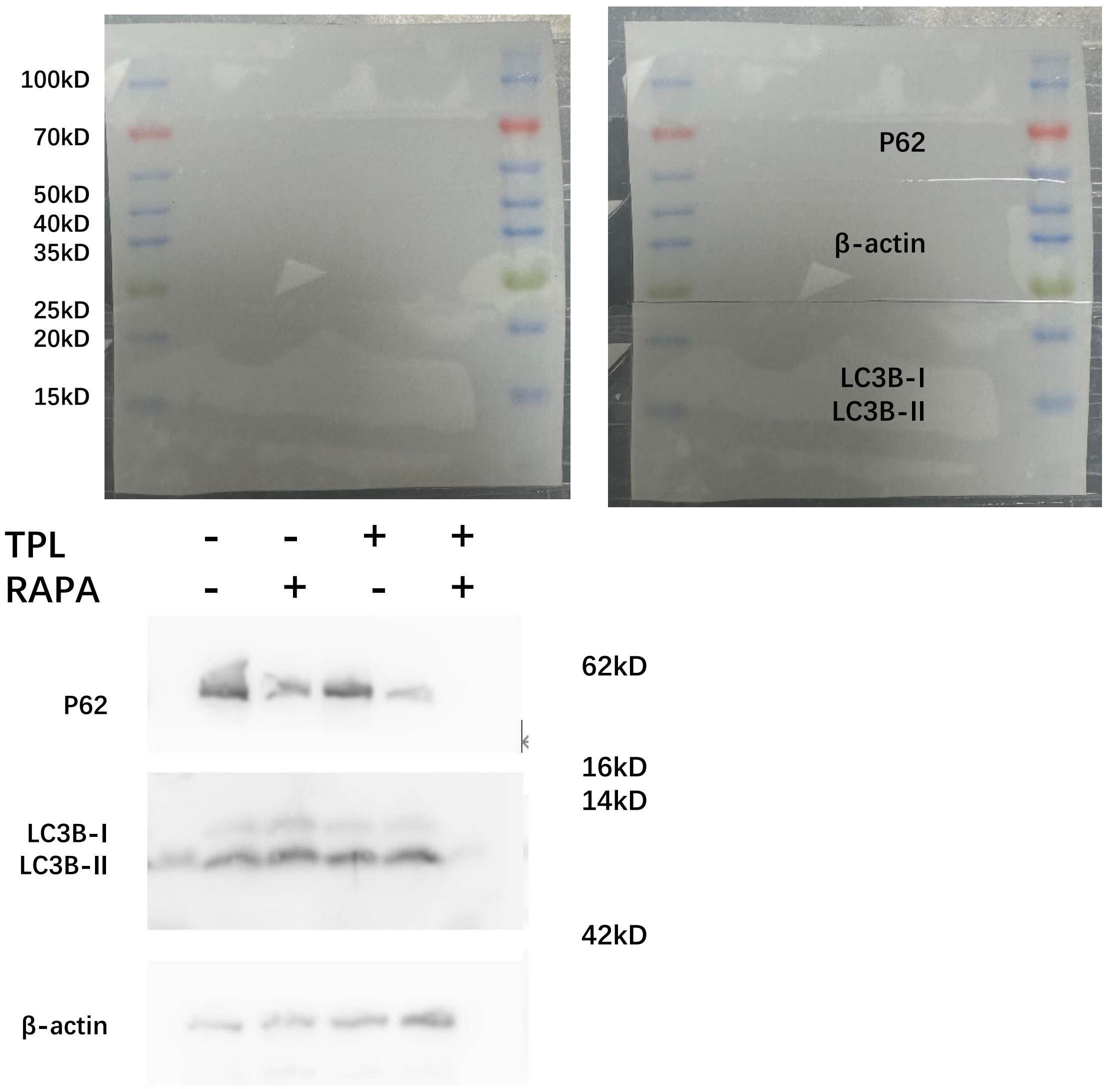

Fig S4-F ①②

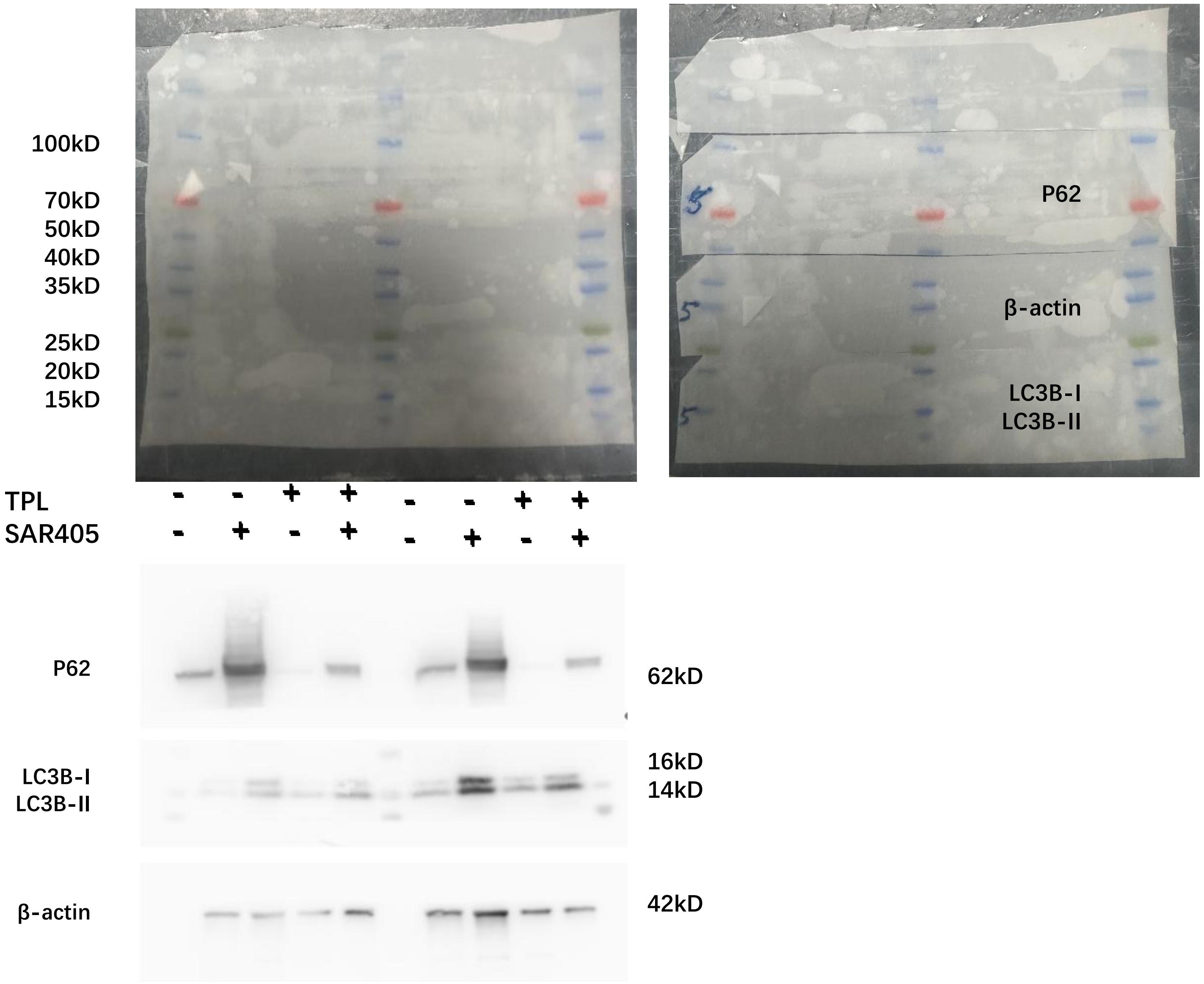

100kD
70kD
50kD
40kD
35kD
25kD
20kD
15kD
P62
β-actin
LC3B-I
LC3B-II
TPL - - + + - - + +
SAR405 - + - + - + - +
P62
62kD
LC3B-I
LC3B-II
16kD
14kD
β-actin
42kD

Fig S4-F ③④

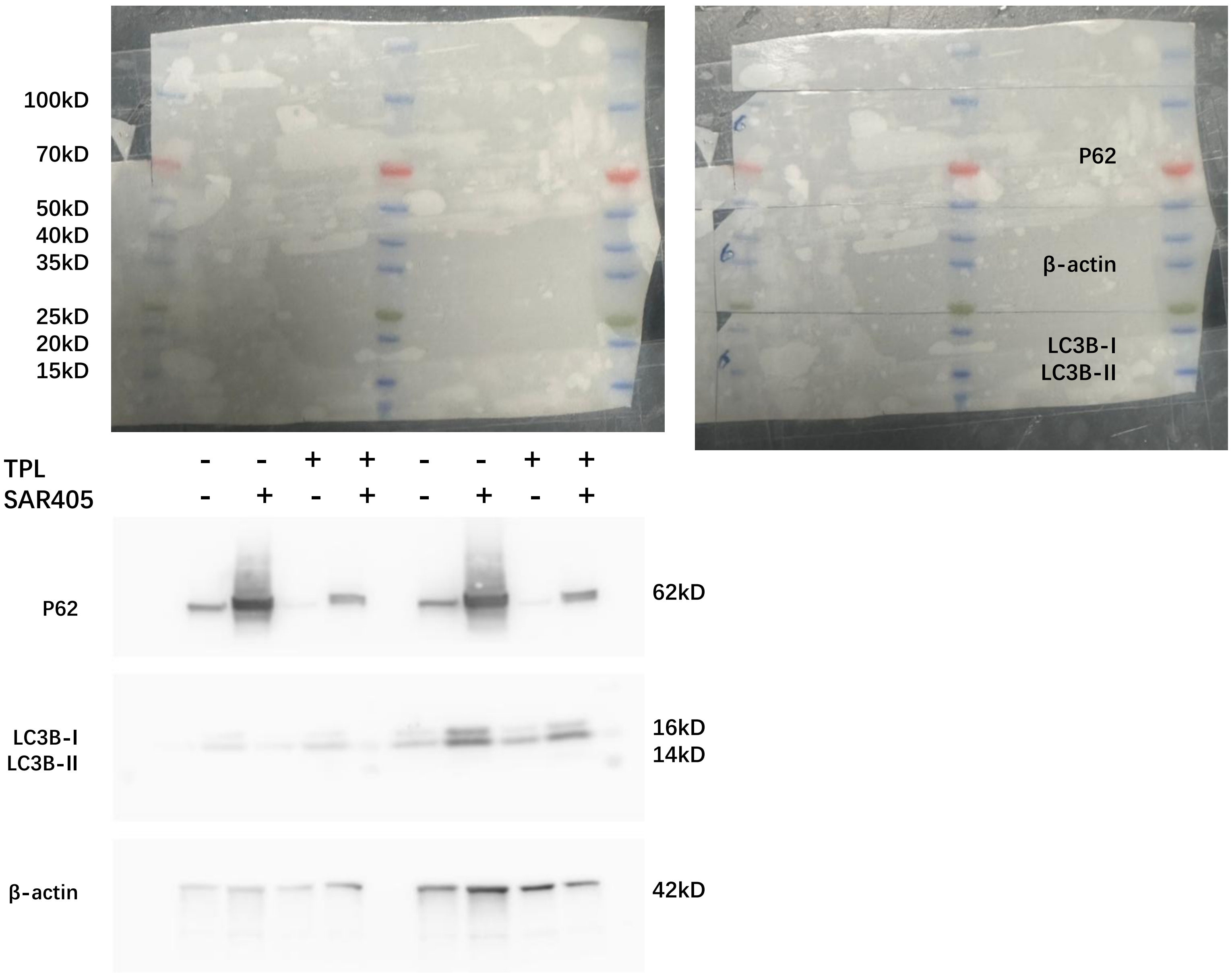
100kD
70kD
50kD
40kD
35kD
25kD
20kD
15kD
P62
β-actin
LC3B-I
LC3B-II
TPL - - + + - - + +
SAR405 - + - + - + - +
P62
62kD
LC3B-I
LC3B-II
16kD
14kD
β-actin
42kD

Fig S5-C ①②

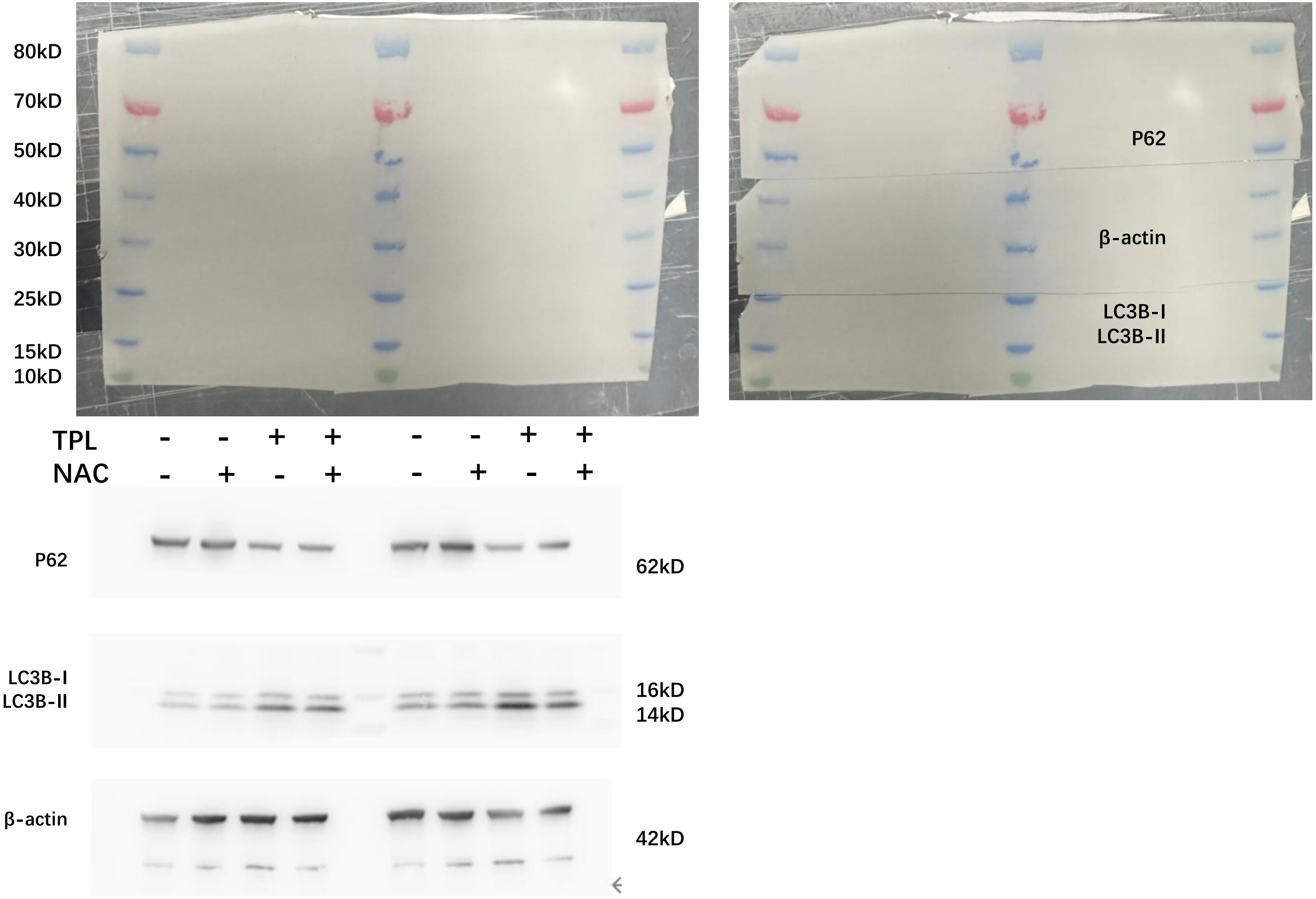

80kD
70kD
50kD
40kD
30kD
25kD
15kD
10kD
P62
β-actin
LC3B-I
LC3B-II
TPL - - + + - - + +
NAC - + - + - + - +
P62
62kD
LC3B-I
LC3B-II
16kD
14kD
β-actin
42kD

Fig S5-C ③④

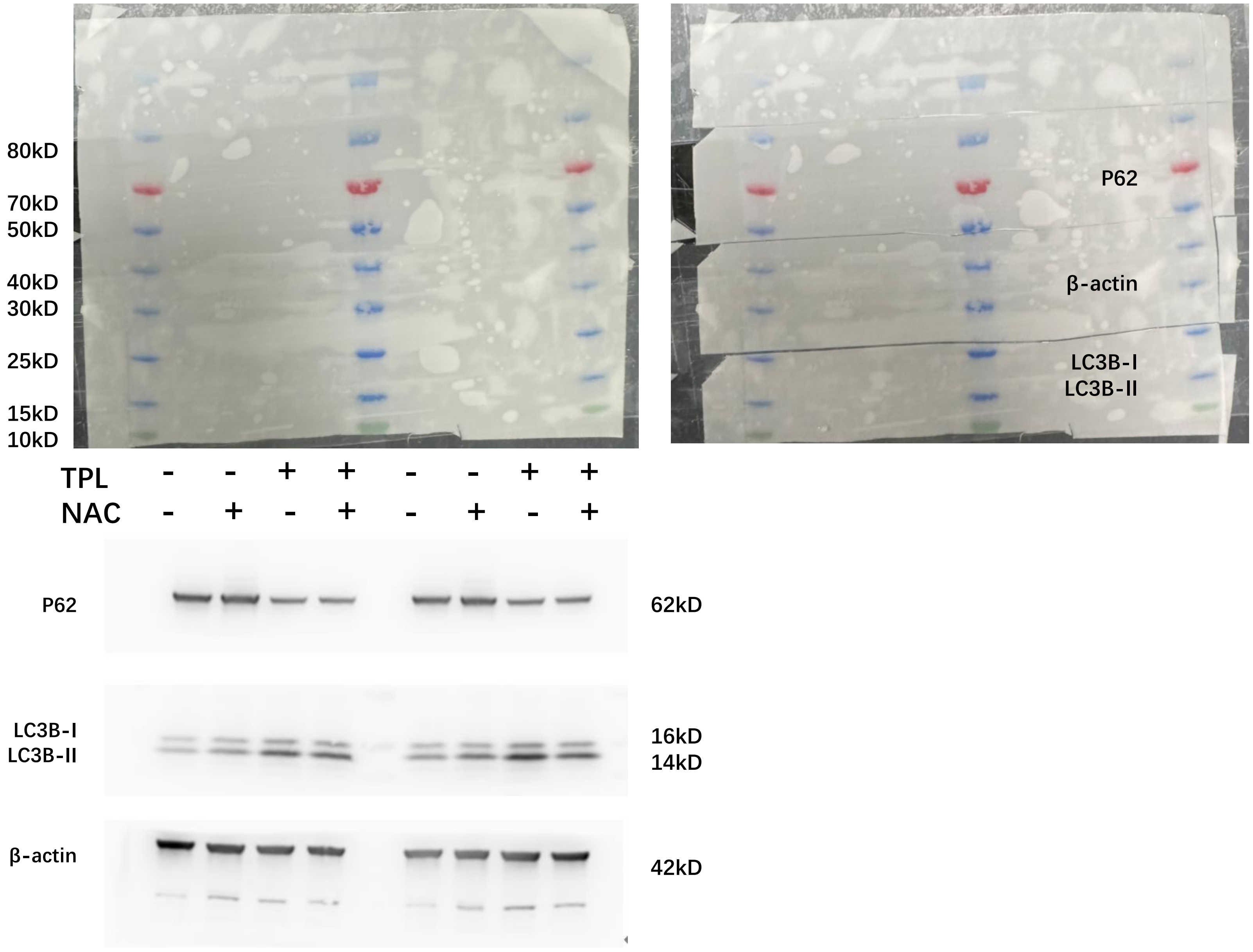
80kD
70kD
50kD
40kD
30kD
25kD
15kD
10kD
P62
β-actin
LC3B-I
LC3B-II
TPL - - + + - - + +
NAC - + - + - + - +
P62
62kD
LC3B-I
LC3B-II
16kD
14kD
β-actin
42kD
